# Recombinant human OVGP1 increases intracellular calcium and further potentiates the effects of progesterone on human sperm

**DOI:** 10.1007/s10815-022-02591-0

**Published:** 2022-08-16

**Authors:** Sydney C. Vanderkooi, Yuewen Zhao, Patricia D. A. Lima, Frederick W. K. Kan

**Affiliations:** 1 Department of Biomedical and Molecular Sciences, Faculty of Health Sciences, Queen’s University, Kingston, Ontario K7L 3N6 Canada; 2 Yale Fertility Center, Division of Reproductive Endocrinology and Infertility, Department of Obstetrics, Gynecology & Reproductive Sciences, Yale University, Orange, Connecticut 06477 USA; 3 Queen’s CardioPulmonary Unit, Faculty of Health Sciences, Queen’s University, Kingston, Ontario K7L 3N6 Canada

**Keywords:** Human reproduction, Oviduct-specific glycoprotein, Progesterone, Sperm capacitation, In vitro fertilization

## Abstract

**Purpose:**

To investigate the effects of recombinant human oviduct–specific glycoprotein (rHuOVGP1) alone and in combination with progesterone (P4) on intracellular Ca^2+^ concentration [Ca^2+^]_i_ and to investigate if rHuOVGP1 in combination with P4 can further enhance tyrosine phosphorylation (pY) of sperm proteins during human sperm capacitation.

**Methods:**

Fluorometric flow cytometry was performed to examine the effects of rHuOVGP1 on [Ca^2+^]_i_ in human sperm during capacitation. Confocal microscopy was used in conjunction with live cell imaging to analyze the influence of rHuOVGP1 and P4 on [Ca^2+^]_i_ in the sperm tail and to examine the involvement of CatSper channels in their effect on [Ca^2+^]_i_. Western blot analysis was performed to assess the protein levels of p105, a major tyrosine-phosphorylated sperm protein.

**Results:**

rHuOVGP1 increases [Ca^2+^]_i_ in human sperm at the beginning of capacitation and further increases and sustains the level of [Ca^2+^]_i_ in the sperm tail following the addition of P4. Inhibition of CatSper channels impedes the effects of rHuOVGP1 on [Ca^2+^]_i_ in the sperm tail. P4 alone can increase pY of a major human sperm protein, p105, yet yields a further increase when used in combination with rHuOVGP1.

**Conclusion:**

The present study revealed that rHuOVGP1 may work with P4 to upregulate [Ca^2+^]_i_ at the beginning of capacitation in part through CatSper channels which, in turn, leads to the downstream event of pY of sperm proteins and enhancement of sperm capacitation.

**Supplementary information:**

The online version contains supplementary material available at 10.1007/s10815-022-02591-0.

## Introduction

The mammalian oviductal fluid within the lumen of the oviduct is essential for gamete transport and maturation, sperm selection and capacitation, fertilization, early embryonic development, and transport of the embryo to the uterus [1]. It was first discovered in 1951 that sperm that have reached and resided in the oviduct acquire the full fertilizing capacity [2]. The oviductal non-ciliated secretory cells synthesize and secrete a major glycoprotein known as oviductin, or oviduct-specific glycoprotein (OVGP1) [3, 4]. This glycoprotein has been identified in hamsters [5, 6], mice [7], rabbits [8, 9], baboons [10], rhesus monkeys [11], sheep [12], pigs [13], cows [14], goats [15], cats [16], dogs [4], and humans [17]. Results derived mainly from mammalian in vitro functional studies have shown that OVGP1 is able to bind to sperm [18, 19] and the zona pellucida of oocytes [20–22], increase sperm motility [19], sperm capacitation [23], sperm-egg binding and penetration rates [18, 22], decrease polyspermy [15, 21], and enhance embryo quality and development [24–26]. Our laboratory has successfully produced, for the first time, recombinant human OVGP1 (rHuOVGP1) which can bind to human sperm and oocytes, enhance sperm capacitation by the increase in the level of protein tyrosine phosphorylation, potentiate acrosome reaction, and increase sperm-egg binding [27, 28]. However, the functional mechanism of how rHuOVGP1 enhances tyrosine phosphorylation of sperm proteins is not fully understood and needs to be further investigated.

The changes associated with capacitation are modulated by components of the oviductal fluid [29]. One of the initial changes described in capacitating sperm is the influx of Ca^2+^ ions [30]. The cation channels of sperm (CatSper), located in the principal piece of the sperm tail control the entry of Ca^2+^ ions into sperm, and they are required for the hyperactivation of sperm motility and male fertility [31]. A large number of Ca^+2^ permeable ion channels has been reported in sperm, including high voltage–activated and low voltage–activated (T-type) Ca^2+^-selective channels, transient receptor potential (TRP) channels, cyclic nucleotide–gated (CNG) channels, and cation channels of sperm (CatSper) [32]. Among these Ca^+2^ permeable ion channels, the CatSper family of channels is the only family of ion channels that is considered functionally significant for sperm physiology in regulating the influx of Ca^2+^ in sperm that is critical for sperm fertility. Experiments carried out with targeted disruption of CatSper in the mouse provide evidence that CatSper is required for normal sperm motility, penetration of the egg, cAMP-induced Ca^2+^ influx, and male fertility [31]. Experiments carried out with indirect immunofluorescence and immunogold electron microscopy in the latter study localized sperm CatSper predominantly to the principal piece of the sperm tail with a weak labeling over the sperm head and mid-piece [31].

After ovulation, the cumulus cells that surround the oocyte secrete the sex hormone progesterone (P4) [30, 33]. In human sperm, progesterone has been shown to stimulate an increase in Ca^2+^ by a non-genomic mechanism [33, 34]. Evidence has shown that P4 binds to the P4 receptor on the plasma membrane of sperm and activates the CatSper channels, thus inducing the influx of Ca^2+^ that initiates a cascade of signal transduction events that subsequently lead to sperm capacitation [31, 35]. The link between CatSper-mediated Ca^2+^ signaling and tyrosine phosphorylation of sperm proteins in mice was investigated by Chung and co-workers [36] where they demonstrated that CatSper organizes Ca^2+^ signaling domains that orchestrate the timing and extent of tyrosine phosphorylation cascade. The latter research suggests that disruption of the CatSper channel complex downregulates the capacitation-initiated tyrosine phosphorylation pathway [36].

The level of protein tyrosine phosphorylation appears to increase in a dose-dependent manner in response to P4 over the first hour of human sperm capacitation, yet this effect can be reduced when sperm are treated with a non-selective Ca^2+^ blocker [37]. These results further imply a connection between Ca^2+^ signaling and tyrosine phosphorylation during sperm capacitation. With the information above outlining the interplay between sperm protein tyrosine phosphorylation, P4, Ca^2+^ influx, and CatSper channels, as a first step to better understand the mechanism regulating rHuOVGP1-enhanced tyrosine phosphorylation of sperm proteins during sperm capacitation, the aim of the present study was to examine the effects of rHuOVGP1 alone and in combination with progesterone on Ca^2+^ influx and tyrosine phosphorylation of sperm proteins during capacitation.

## Materials and methods

### Materials

The following chemical reagents and materials were obtained from the sources indicated: Percoll, Fluo3, 7-aminoactinomycin D (7AAD), and goat anti-mouse IgG-HRP antibody (Thermo Fisher Scientific, Waltham, MA, USA); HC-056456 (3,4-Bis(2-thienoyl)-1,2,5-oxadiazole-N-oxide) (Cedarlane, Burlington, ON, Canada); monoclonal mouse anti-phosphotyrosine antibody (Cat#P5872), monoclonal mouse anti-α-tubulin antibody (Cat#T6199), and concanavalin A (Con-A; Cat#C5275) (Sigma, St. Louis, MO, USA); progesterone (Life Technologies, Carlsbad, CA, USA); protein ladder (FroggaBio, Concord, ON, Canada); clarity Western enhanced chemiluminescence (ECL) substrate (Bio-Rad Laboratories Inc., Hercules, CA, USA). All other chemicals were obtained from Sigma (St. Louis, MO, USA).

The rHuOVGP1 used in the present study was previously prepared in our laboratory. The secretory form of rHuOVGP1 was produced from HEK293 cells using recombinant DNA technology as described in detail elsewhere [27]. rHuOVGP1 was purified using ammonium sulfate precipitation and gel filtration chromatography. The resulting rHuOVGP1 showed a single band corresponding to the 120–150 kDa size range of native human OVGP1, and its identity as human OVGP1 was further confirmed by mass spectrometry [27].

### Preparation of sperm from fresh human semen samples

The present study was approved by the Queen’s University Health Sciences & Affiliated Teaching Hospitals Research Ethics Board. Human semen samples were obtained from Flow Labs Kingston, located in Kingston General Hospital (Kingston, ON, Canada), via consenting donors by masturbation after 2 to 3 days of abstinence. Upon collecting the samples, they were liquefied in a 35 °C water bath. The volume and viscosity of the semen as well as the sperm motility were assessed according to guidelines of the World Health Organization [38]. To prepare the sperm samples for subsequent experiments, liquefied semen was layered on top of a Percoll gradient made up of 2-mL fractions each of 40% and 65% and 1 mL of 95% Percoll, followed by centrifugation at 1000 × *g* for 30 min at ambient temperature to isolate sperm from seminal fluid. Percoll was prepared in 1X HEPES-buffered saline (HBS; 25 mM HEPES, 130 mM NaCl, 4 mM KCl, 0.5 mM MgCl_2_, 14 mM D-fructose, pH 7.6). After centrifugation, sperm from the 65–95% interface and within the layer of 95% Percoll were collected and used for subsequent experiments, as they represent a highly motile population. In general, sperm which are most motile are indicative of better quality and viability. Sperm were then washed with 10 mL of HBS and centrifuged at 500 × *g* for 10 min at ambient temperature. The supernatant was removed, and sperm concentration was then determined using a hematocytometer counter chamber device.

### Preparation of sperm for assessment of [Ca^2+^]_i_

Fluo3 is a fluorescence indicator of intracellular calcium. The Fluo3 stock solution was purchased as 1 mg in powder form and prepared at a concentration of 1 mM in DMSO. Once sperm cells were counted, they were added at a concentration of 1 × 10^7^ cells/mL into the non-capacitating medium (120 mM NaCl, 4.8 mM KCl, 1.2 mM KH_2_PO_4_, 1.2 mM MgSO_4_, 0.25 mM Na pyruvate, 21.6 mM Na lactate, 10 mg/L phenol red, 5.6 mM d-glucose, 10 mM HEPES, pH 7.4) in order to prevent the onset of capacitation during preparation. Subsequently, the sperm cell solution was supplemented with Fluo3 (2 mM) and incubated at 37 °C with 5% CO_2_ for 30 min. The rest of the cells were added to the non-capacitating medium at a concentration of 1 × 10^7^ cells/mL without Fluo3 for subsequent use as controls. At the end of incubation, cells were removed from the incubator (Sanyo MCO-17AIC CO_2_ incubator, Marshall Scientific, Hampton, NH, USA) and centrifuged at 1000 × *g* for 5 min at ambient temperature. Sperm were then washed with 1 mL of HBS and centrifuged again. The supernatant was removed, and the remaining volume was measured.

### Flow cytometry

Following preparation for assessment of [Ca^2+^]_i_ with Fluo3, sperm cells at a concentration of 4 × 10^6^ cells/mL were added to 500 mL of modified Biggers-Whitten-Wittingham medium (BWW; 94.6 mM NaCl, 4.8 mM KCl, 1.7 mM CaCl_2_, 1.2 mM KH_2_PO_4_, 1.2 mM MgSO_4_, 0.25 mM Na pyruvate, 21.6 mM Na lactate, 10 mg/L phenol red, 25.1 mM NaHCO_3_, 5.6 mM d-glucose, 10 mM HEPES, pH 7.4) supplemented with 0.3% BSA to enable sperm capacitation. The indicator 7-Aminoactinomycin D (7AAD) (2 mL per 2 × 10^6^ sperm cells) was added to all experimental reactions to assess sperm viability prior to data acquisition. Intracellular Ca^2+^ was then assessed using flow cytometry (FL2 525/50; SH800S, Sony, SanJose, USA). Following 3 min of baseline fluorescence reading, rHuOVGP1 (25, 50, 75, and 100 mg/mL) was added, respectively, to the cell suspension (4 × 10^6^ cells/mL), and the data were acquired for further 10 min. As controls, the fluorescent intensity of unstained non-capacitated sperm cells (4 × 10^6^ cells/mL) as well as individual stained cells were measured. Data were analyzed using FlowJo (Ashland, USA), and the median of Fluo3 fluorescence was recorded as a measurement of intracellular Ca^2+^.

### Calcium imaging

Sperm cells were prepared for assessment of [Ca^2+^]_i_ with Fluo3 as described above. Sperm cells (2 × 10^6^ cells/mL) in non-capacitating medium were plated in 35-mm dishes (No. 1.5 Coverslip; 10 mm Glass Diameter; MatTeK Life Sciences) pre-coated with Concanavalin A (a carbohydrate-binding protein; 1 mg/mL in PBS) as previously described [39]. Sperm cells were imaged using a live cell imaging system, the OkoLab stage-top microscope incubator (OkoLab Bold Line, Pozzuoli, Italy) connected to the Leica TCS SP8 confocal microscope (Leica Microsystems, Wetzlar, Germany). Sperm cells were imaged for 1 min in capacitating (BWW) medium to obtain baseline measurements, followed by subsequent imaging after treatment with rHuOVGP1 (100 mg/mL), the CatSper channel blocker, HC-056456 (3 or 10 mM), and progesterone (P4; 1 mM). The aforementioned various experimental groups are summarized in the chart below:
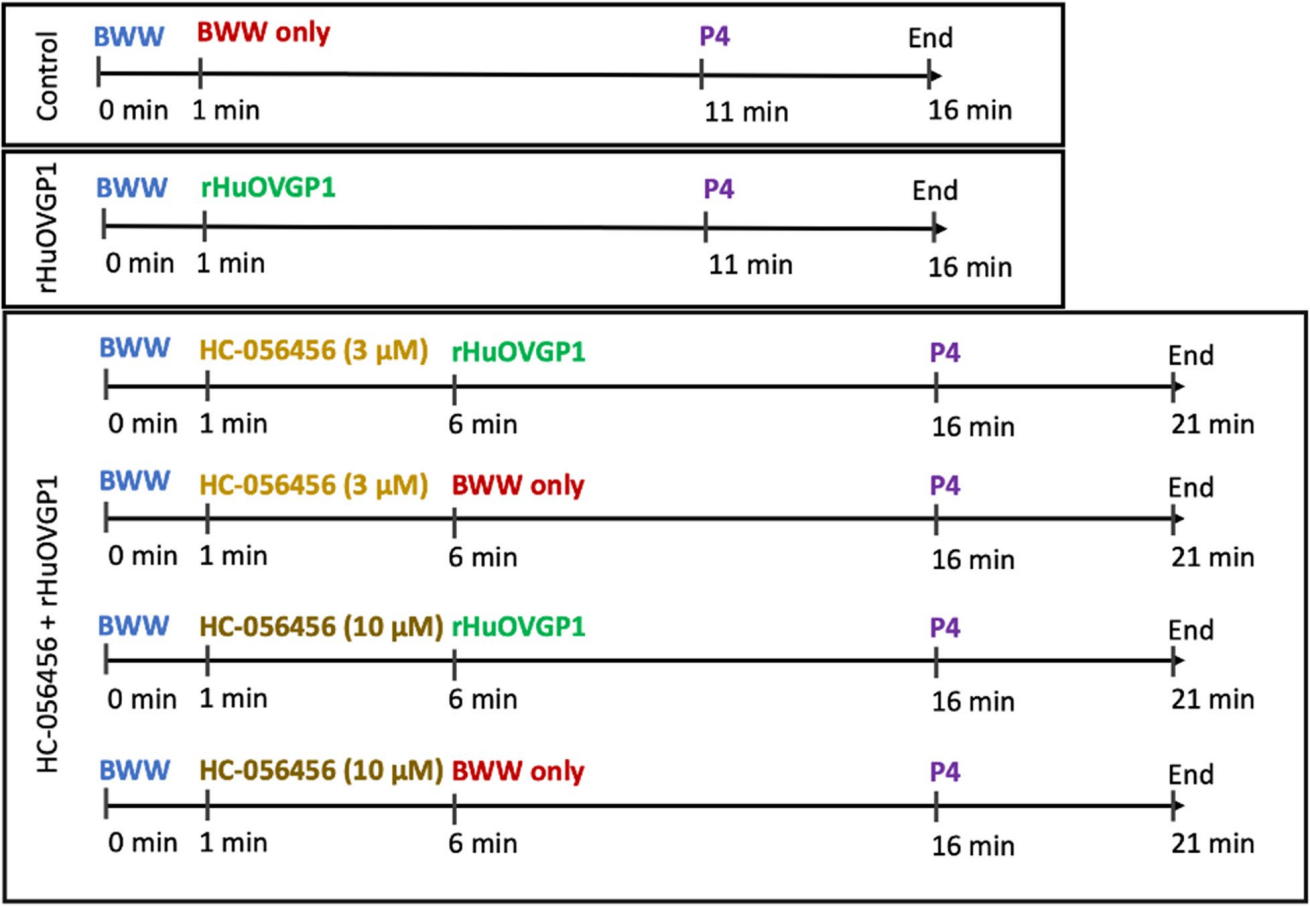


### Western blot analysis

Our laboratory has previously reported that the tyrosine phosphorylation level of p105, a major human sperm protein, can be further enhanced in the presence of rHuOVGP1 at an optimal concentration of 50 mg/mL in capacitating medium [27]; therefore, rHuOVGP1 at a concentration of 50 mg/mL was used in the present study to assess the effect of rHuOVGP1 on tyrosine phosphorylation of p105 by Western blot. Aliquots of the sperm (2 × 10^7^ cells/mL) were prepared in the absence or presence of 50 mg/mL of rHuOVGP1 with different concentration of P4 (0 nM, 1 nM, 10 nM, 100 nM, 1 mM, or 10 mM). These samples were capacitated via incubation at 37 °C with 5% CO_2_ in modified Biggers-Whitten-Wittingham medium supplemented with 0.3% BSA for different time-points (0–4 h). The capacitating medium was pre-equilibrated overnight at 37 °C with 5% CO_2_. During the incubation period, samples were mixed gently every 15 min to resuspend the cells. At the end of the incubation period, the samples were washed with 1 mL of 1X HBS and centrifuged at 1000 × *g* for 5 min at room temperature. The sperm samples were vortexed for 30 s in sample buffer (6% SDS, 30% glycerol, 12% b-mercaptoethanol, 0.02% bromophenol blue, 6 mM Na_3_VO_4_, 187.5 mM Tris–HCl, pH 6.8) and boiled for 5 min to denature the proteins, following by centrifugation (16,000 × *g* for 5 min at room temperature). Samples were loaded into wells in the gel apparatus. Sperm proteins were separated by SDS-PAGE (SDS polyacrylamide gel electrophoresis) and transferred onto polyvinylidene difluoride (PVDF).

### Evaluation of tyrosine phosphorylation of sperm proteins

As tyrosine phosphorylation of sperm proteins is a biochemical hallmark of sperm capacitation, changes in the level of tyrosine phosphorylation of sperm proteins were determined by Western blot analysis. The non-specific binding on the PVDF membrane was blocked with 5% milk in 1X Tris-buffered saline (150 mM NaCl, 50 mM Tris, pH 7.5) containing 0.05% Tween 20 (TBST) for 45 min. The PVDF membrane was incubated in the cold room for 1–2 h with a monoclonal mouse anti-phosphotyrosine antibody (0.1 mg/mL in TBST). The membranes were washed and incubated with goat anti-mouse IgG-HRP antibody (diluted in blocking solution at 0.02 mg/mL) for 1 h at room temperature. After being washed, the membrane was placed in ECL (enhanced chemiluminescence) which detects horseradish peroxidase (HRP) and allows for visualization of the protein bands of interest. The same membrane was probed with α-tubulin (monoclonal mouse anti-α-tubulin antibody; 0.1 mg/mL in TBST) for 1 h and goat anti-mouse IgG-HRP antibody (diluted in blocking solution at 0.02 mg/mL) for 1 h at room temperature as loading control, and to normalize the data.

### Statistical analyses

In all experimental groups and controls, a total of 50 to 100 sperm cells in each individual group were examined and assessed. Statistical tests were performed using Prism 9 (GraphPad Software, San Diego, CA, USA). Changes of [Ca^2+^]_i_ were determined by measuring the fluorescence intensity of Fluo3. The assessment of [Ca^2+^]_i_ using flow cytometry for each experimental sample was performed by comparing the baseline fluorescence (− 200 s) with the fluorescence at further times-point following the addition of rHuOVGP. The data were expressed as a fold change using two-way ANOVA with Dunnett’s post hoc test. The comparison of the intensity of fluorescent staining between rHuOVGP1-treated cells at different concentrations of rHuOVGP1 and the untreated cells at a specific time-point was analyzed using Student’s *t* tests.

The assessment of [Ca^2+^]_i_ using live cell imaging was performed at the 16- (Fig. 2) and 21-min (Fig. 3) incubation periods. As the sperm cell fluorescence was recorded every ~ 1.5 s, the analysis consisted of averaging a sequence of 45 to 65 recorded time-points to obtain a final set of 15 (Fig. 2) and 20 (Fig. 3) time-points. The data were expressed as a fold increase from the initial fluorescent intensity. The comparison of the intensity of fluorescent staining between the cells treated with rHuOVGP1 and the untreated cells at a specific time-point was analyzed using Student’s *t* tests.

For assessment of protein tyrosine-phosphorylation via Western blot analysis, the band intensities were quantified by densitometry using ImageJ software (National Institutes of Health, Bethesda, MD, USA). The intensities of the bands were normalized to those of α-tubulin. The level of tyrosine phosphorylation of sperm proteins at each time-point was compared to the 0-h time-point and expressed as a fold increase using two-way ANOVA with Dunnett’s post hoc test. The comparison between treated cells and untreated cells at a specific time-point was analyzed using Student’s *t* tests. *P* values of < 0.05 were considered significant.

## Results

### Effects of rHuOVGP1 on [Ca^2+^]_i_ of human sperm

**Fig. 1:**
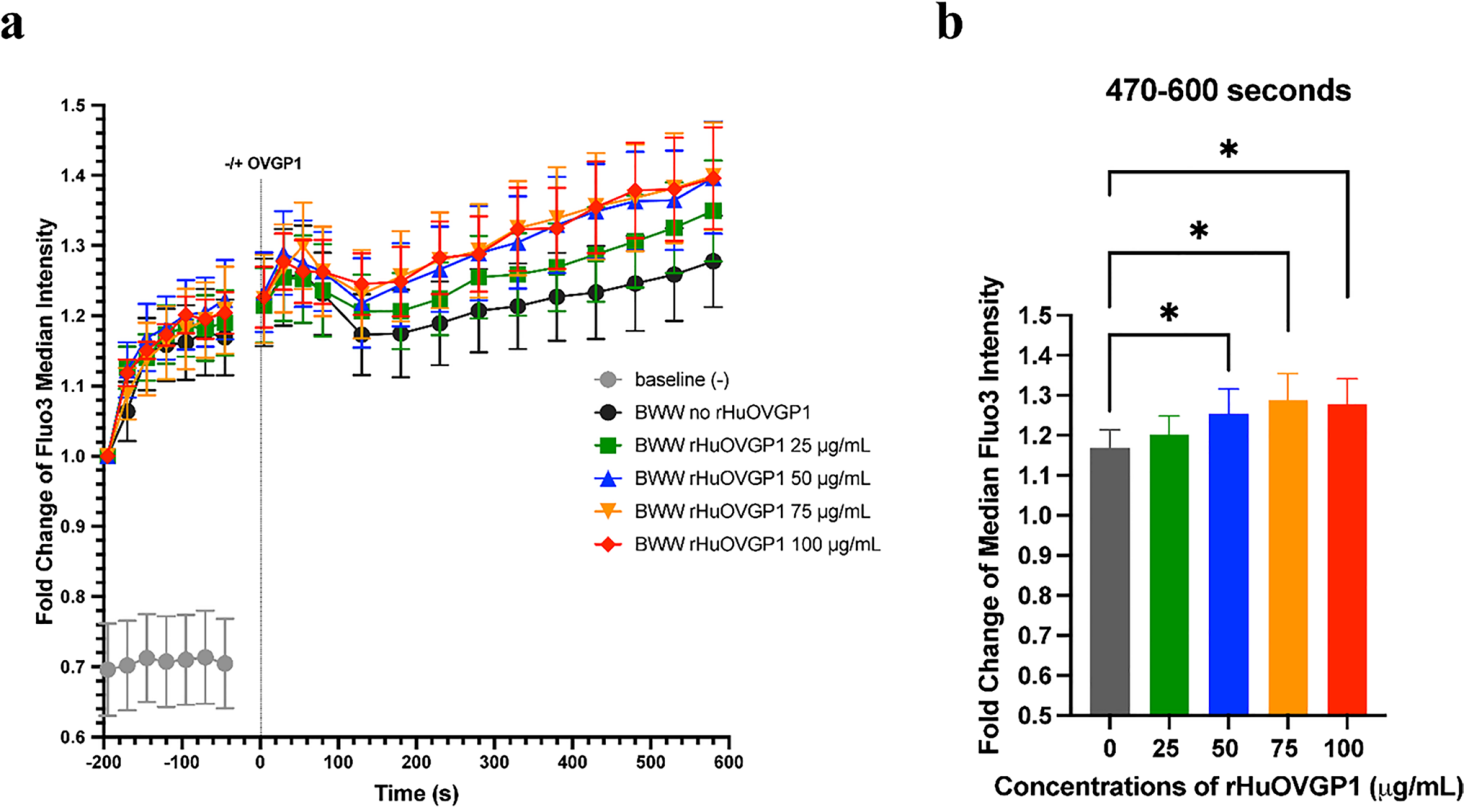
rHuOVGP1 increases [Ca^2+^]_i_ in human sperm at the beginning of capacitation. **a** Line graph of the median fluorescent intensity of Fluo3 from the flow cytometry analysis of [Ca.^2+^]_i_ in sperm. Following 3 min (shown as − 200 to − 20 s) of baseline fluorescence reading, rHuOVGP1 (0, 25, 50, 75, and 100 mg/mL) was added, respectively, to the cell suspension (time = 0 s), and the data were acquired for a further 10 min. **b** Histogram of the mean fluorescent intensity over the period of 470–600 s after the addition of rHuOVGP1 (0, 25, 50, 75, and 100 mg/mL). Data are represented as fold of change in fluorescent labeling + / − SEM; *n* = 7 patients; *p < 0.05

Due to the shared localization of CatSper channels and tyrosine phosphorylation of sperm proteins in the principal piece of the sperm tail [28, 36], we sought out to investigate the effects of OVGP1 on [Ca^2+^]_i_ during capacitation. Flow cytometry experiments were carried out where sperm samples were incubated in capacitating medium followed by the addition of different concentrations of rHuOVGP1 (0, 25, 50, 75, or 100 mg/mL) at *t* = 0 for 10 min. Additionally, a sample incubated in non-capacitating medium was analyzed to act as a baseline for fluorescent intensity. The level of [Ca^2+^]_i_ in sperm, indicated by the intracellular calcium indicator Fluo3, appeared to gradually increase when sperm cells were exposed to capacitating (BWW) medium (Fig. 1a). However, the level of [Ca^2+^]_i_ appeared to be further increased when sperm were subjected to treatment with increasing concentrations of rHuOVGP1 (Fig. 1a). The level of [Ca^2+^]_i_ over the period of 470–600 s after the addition of rHuOVGP1 is significantly higher in sperm treated, respectively, with 50, 75, and 100 mg/mL of rHuOVGP1 compared to untreated sperm (Fig. 1b). The table in Online Resource 1 displays the results from a two-way ANOVA statistical analysis comparing the level of Fluo3 median intensity over time to that of the time-point at 45 s prior to the addition of rHuOVGP1 indicated as − 45 s. The results showed that sperm [Ca^2+^]_i_ was significantly increased at earlier time points when sperm were treated with higher doses of rHuOVGP1 during the 10-min incubation period. Treating sperm with rHuOVGP1 significantly increased the level of intracellular calcium in a time-dependent manner. Overall, these results indicated that the presence of OVGP1 increases [Ca^2+^]_i_ at the beginning of sperm capacitation and maintains this increase in [Ca^2+^]_i_ over the 10-min time course.

### Effects of treating sperm with rHuOVGP1 in combination with P4 on [Ca^2+^]_i_

**Fig. 2:**
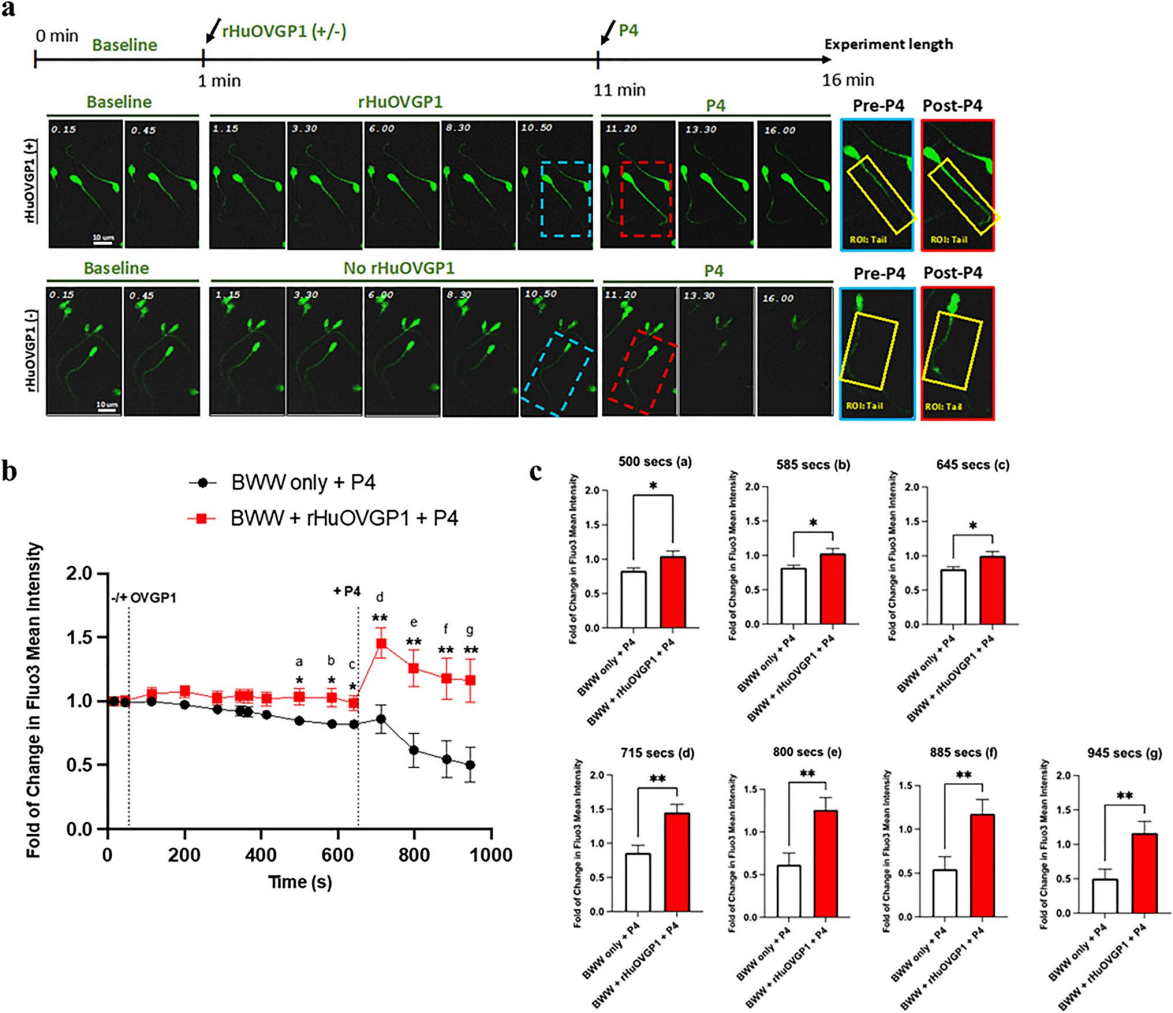
Treatment of sperm with rHuOVGP1 further increases and sustains the level of [Ca^2+^]_i_ in human sperm tail following treatment with P4. **a** Representative images of calcium live cell imaging experiments with or without rHuOVGP1 (100 mg/mL) treatment. High magnifications of sperm cells indicated by blue (pre-P4) and red (post-P4) dashed boxes are shown on the right side of the figure with regions of interest (ROIs) of sperm tails outlined in yellow. Sperm cells were imaged for 1 min in BWW to obtain baseline measurements, followed by subsequent imaging the same cells treated with or without rHuOVGP1 (100 mg/mL) for 10 min and progesterone (P4; 1 mM) for 5 min. **b** Line graph of the relative mean fluorescent intensity of Fluo3 from the live cell imaging analysis of [Ca^2+^]_i_ in sperm tails treated with or without rHuOVGP1 (100 mg/mL) followed by the treatment with P4 (1 mM). **c** Histograms of the relative mean fluorescent intensity from [Ca.^2+^]_i_ analysis for sperm incubated with or without rHuOVGP1 at various time-points (500 (a), 585 (b), 645 (c), 715 (d), 800 (e), 885 (f), and 945 (g) s). Data are represented as fold of change in fluorescent labeling + / − SEM; *n* = 6 patients; *p < 0.05, **p < 0.01

The effects of OVGP1 on [Ca^2+^]_i_ were further studied using fluorometric confocal microscopy with live cell imaging. Sperm cells were imaged in capacitating (BWW) medium to obtain baseline measurements (1 min). Following baseline measurements in BWW, the same cells were treated in the presence or absence of rHuOVGP1 (100 mg/mL) with live cell imaging continued being recorded for 10 min and subsequently treated with P4 (1 mM) for 5 min. Representative images of the live cell imaging over time are shown in Fig. 2a. Sperm cells treated with rHuOVGP1 showed an increasing trend in the level of intracellular calcium compared to untreated cells (Fig. 2b, c: time-points a-c). As expected, based on evidence that P4 induces calcium influx in sperm [40–42], the addition of P4 alone resulted in an increase in [Ca^2+^]_i_ followed by a gradual decrease (Fig. 2a, b). However, this increase in [Ca^2+^]_i_ was further enhanced when sperm cells were treated with rHuOVGP1 followed by the addition of P4 (Fig. 2a–c: time-point d). Moreover, the increase in [Ca^2+^]_i_ observed in sperm treated with rHuOVGP1 was sustained over the 5-min period following treatment with P4 (Fig. 2a–c: time-points e–g). These results demonstrated that treating sperm with OVGP1 amplifies the level of intracellular Ca^2+^ in human sperm tail following treatment with P4. Although the present study focused on the sperm tail, the effects of OVGP1 on [Ca^2+^]_i_ were also assessed in the sperm head (Online Resource 2). There was no difference in the level of [Ca^2+^]_i_ in the heads of sperm treated with OVGP1 alone compared with that of the untreated sperm. However, a pattern was observed similar to that of the sperm tail following the addition of P4 where there was a notable increase in [Ca^2+^]_i_ after treatment with P4 (Online Resource 2: time-point a). Yet, this increase in [Ca^2+^]_i_ in the sperm heads was further enhanced when sperm were sequentially treated first with rHuOVGP1 followed by P4 (online Resource 2: time-points b–d). Taken together, the present results showed that treatment of sperm with OVGP1 increases the level intracellular Ca^2+^ in the human sperm tail; yet, OVGP1 is capable of further enhancing the level of intracellular Ca^2+^ throughout the entire sperm when the initial treatment with OVGP1 is followed by P4.

### Inhibition of CatSper channels impedes the effects of rHuOVGP1 on sperm [Ca^2+^]_i_

**Fig. 3:**
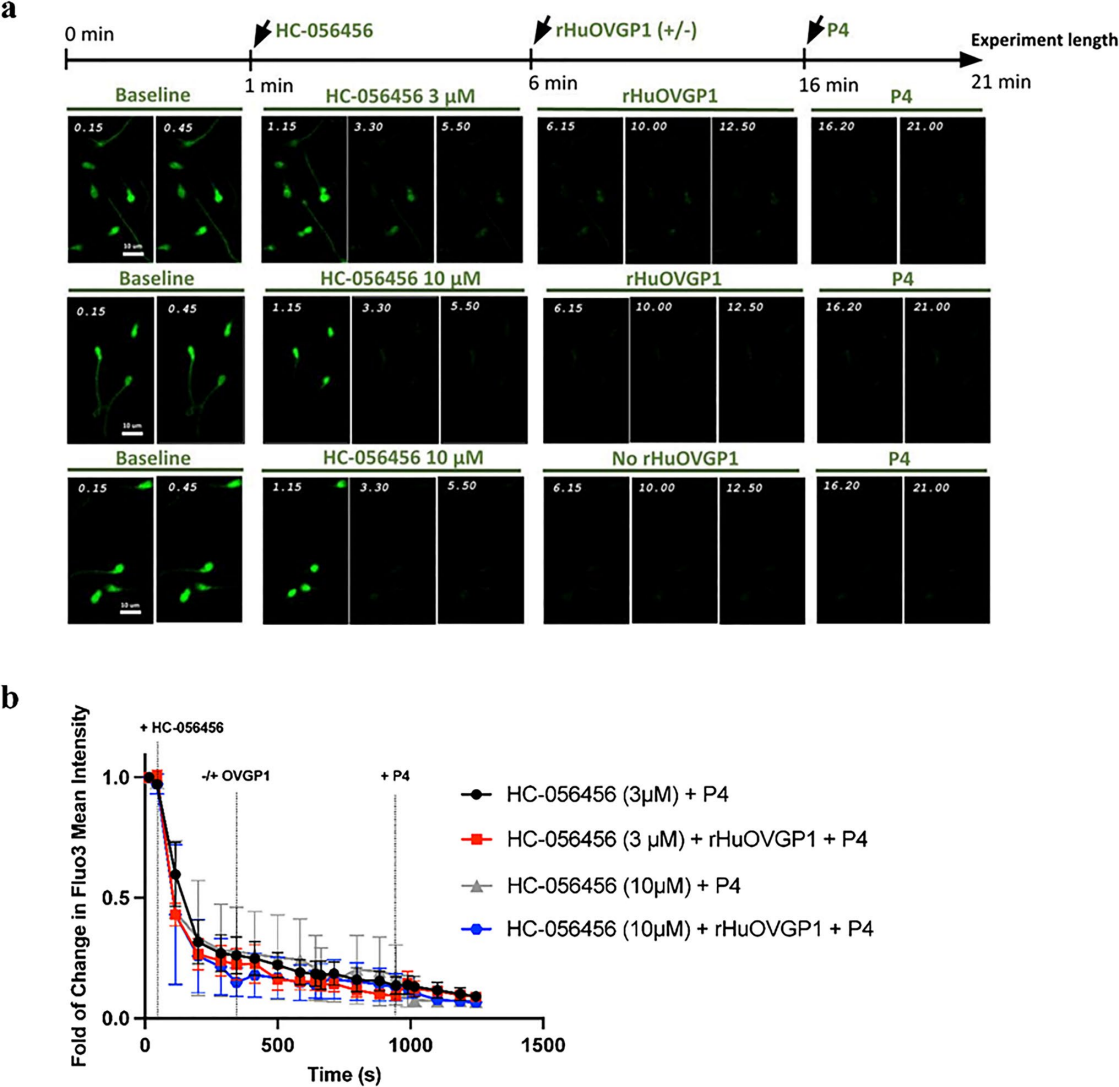
Inhibition of CatSper channels impedes the effects of rHuOVGP1 on [Ca^2+^]_i_ in human sperm tail. **a** Representative images of calcium live cell imaging experiments with or without rHuOVGP1 (100 mg/mL) treatment in the presence of CatSper channel inhibitor HC-056456. Sperm cells were imaged for 1 min in BWW to obtain baseline measurements, followed by the subsequent imaging of the same cells treated with HC-056456 (3 or 10 mM) for 5 min, with or without rHuOVGP1 (100 mg/mL) for 10 min and with progesterone (P4; 1 mM) for 5 min. **b** Line graph of the relative mean fluorescent intensity of Fluo3 from the live cell imaging analysis of [Ca^2+^]_i_ in sperm tails treated with HC-056456 (3 or 10 mM), with or without rHuOVGP1 (100 mg/mL) followed by treatment with P4 (1 mM). Data are represented as fold of change in fluorescent labeling + / − SEM; *n* = 3 patients

CatSper channels play a key role in male fertility by controlling Ca^2+^ entry into sperm [31, 41–44]. To investigate whether rHuOVGP1 increases [Ca^2+^]_i_ through CatSper channels, further live cell imaging was performed using the selective CatSper channel inhibitor HC-056456. The compound HC-056456 has previously been shown to lower the CatSper current in whole cell patch-clamp recordings and reversibly prevent the development of hyperactivated motility during sperm capacitation [43]. Following baseline measurements, live cell imaging of the same cells treated with HC-056456 (at 3 or 10 mM), and with or without rHuOVGP1 (100 mg/mL) were recorded for 10 min and subsequently treated with P4 (1 mM) for 5 min. As anticipated, inhibiting CatSper channels with their specific inhibitor, HC-056456, resulted in a decrease in the level of Fluo3 intensity, indicating a decrease in [Ca^2+^]_i_ (Fig. 3). No increase in [Ca^2+^]_i_ following the addition of P4 was observed in sperm cells pre-treated with HC-056456 alone (Fig. 3). Likewise, no increase in [Ca^2+^]_i_ following the addition of rHuOVGP1 or subsequent addition of P4 was observed in sperm cells pre-treated with HC-056456 (Fig. 3). These results indicated that inhibition of the CatSper channels with HC-056456 impedes the effects of rHuOVGP1 on [Ca^2+^]_i_ in the human sperm tail. As P4 is known to induce calcium influx through CatSper channels [40–42], the present results suggest that OVGP1, like P4, increases intracellular calcium through the CatSper channel.

### Effects of P4 on tyrosine phosphorylation of sperm protein

**Fig. 4:**
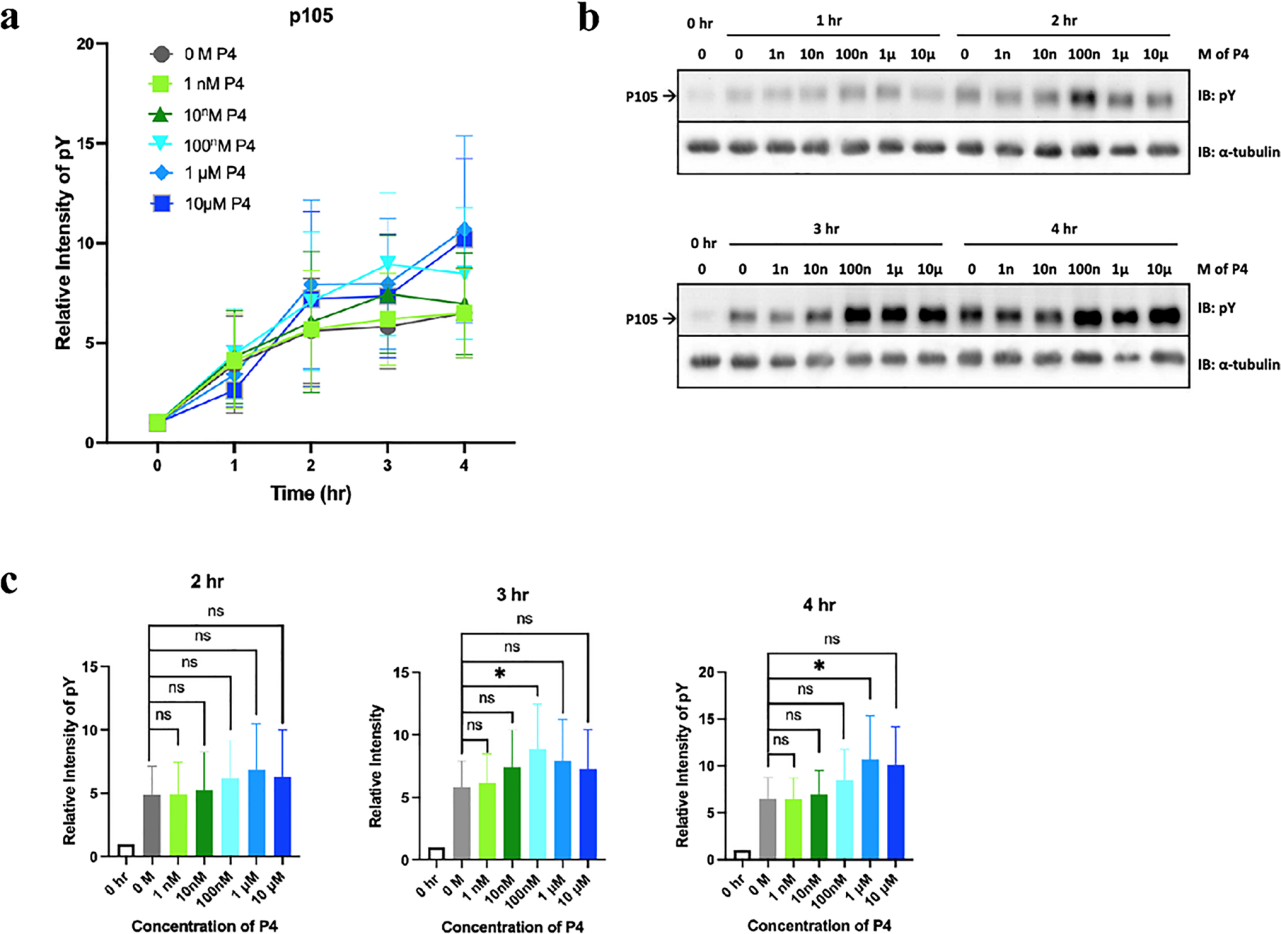
P4 enhances the level of tyrosine phosphorylation of human sperm protein. **a** Line graph showing the effect of different P4 concentrations (0, 1 nM, 10 nM, 100 nM, 1 mM, or 10 mM) on the level of tyrosine phosphorylation (pY) of p105 following 0 to 4 h of capacitation. **b** Representative Western blot analysis showing the effect of different P4 concentrations on the level of tyrosine phosphorylation of p105 following 0 to 4 h of capacitation. **c** Histograms showing the relative intensity of tyrosine phosphorylation (pY) levels of p105 in sperm treated with different P4 concentrations after 2, 3, and 4 h of capacitation. Data are represented as relative intensity of pY + / − SEM; *n* = 6 patients; *p < 0.05; ns: statistically not significant

As time-dependent increase in the level of sperm protein tyrosine phosphorylation is a characteristic feature of sperm capacitation and P4 is known to increase intracellular calcium during sperm capacitation [40, 41], the effects of P4 on enhancing protein tyrosine phosphorylation were assessed. Sperm cells were incubated for 0 to 4 h in capacitating (BWW) medium alone or supplemented with different concentrations of P4 (0 nM, 1 nM, 10 nM, 100 nM, 1 mM, or 10 mM). Our results showed that the most abundantly tyrosine-phosphorylated human sperm protein migrates at 105 kDa (p105), comparable to results obtained from previous studies carried out in our laboratory [27], and was further enhanced by the presence of P4 in the capacitating medium (Fig. 4a, b). As shown in Fig. 4c, the level of tyrosine phosphorylation is further increased with higher concentrations of progesterone. Specifically, the level of tyrosine phosphorylation is significantly increased at 3 h when treated with 100 nM of P4 but is significantly further increased at 4 h when treated with 1 mM of P4 (Fig. 4c). The table in Online Resource 3 displays the results from a two-way ANOVA statistical analysis of tyrosine phosphorylation of each treatment of P4 over time compared to non-capacitated sperm cells. The results showed that the time-points that yielded a significant increase in the level of tyrosine phosphorylation of p105 occurred earlier as the concentration of P4 increased to 1 mM of P4, followed by a later increase in the level of tyrosine phosphorylation at a concentration of 10 mM of P4. Overall, these results showed that P4 can enhance the tyrosine phosphorylation of sperm p105 in a time- and dose-dependent manner and suggest that 1 mM may be the optimal P4 concentration.

### Combined effects of P4 and rHuOVGP1 on tyrosine phosphorylation of sperm protein

**Fig. 5:**
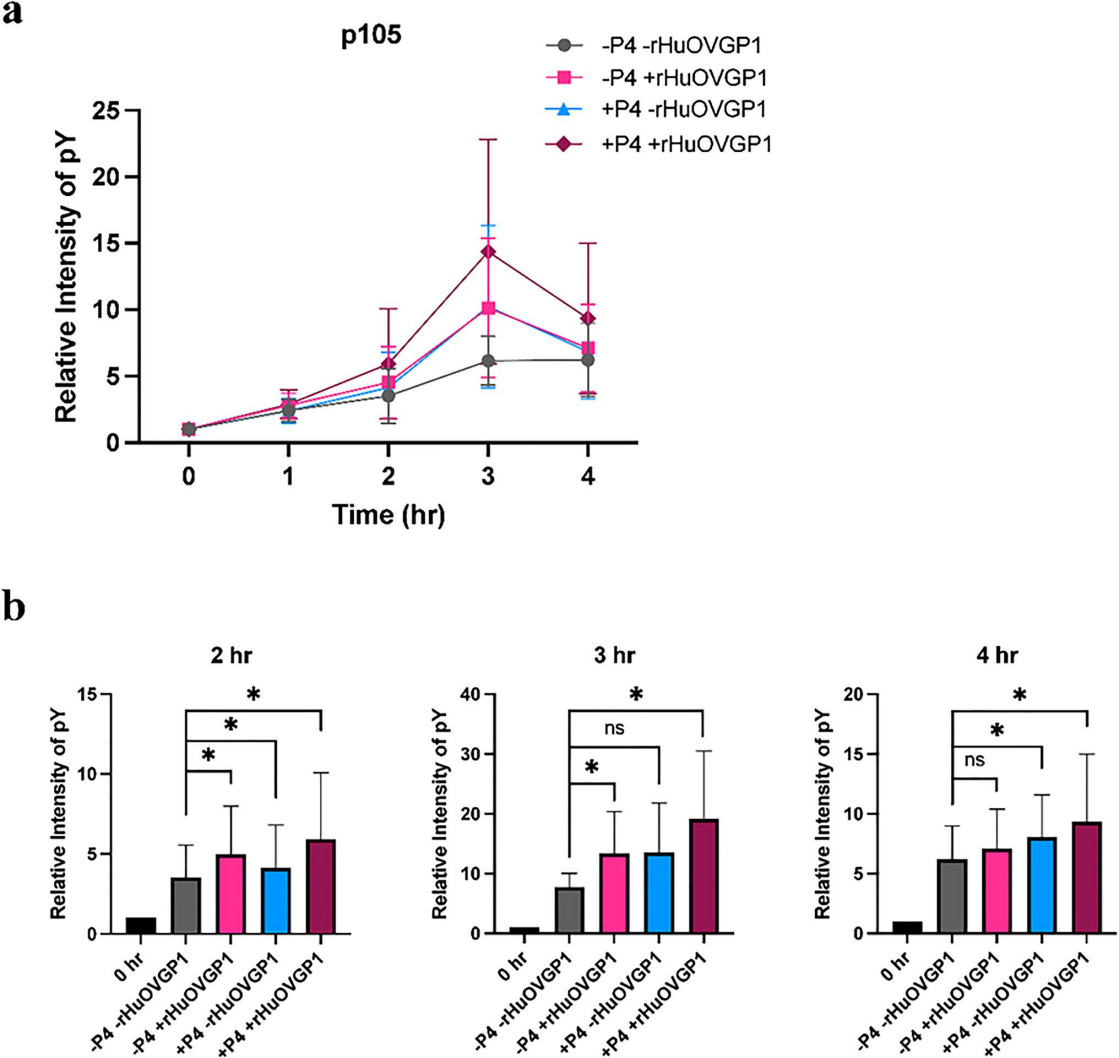
rHuOVGP1 further enhances the positive effects of P4 on the level of tyrosine phosphorylation of protein in human sperm. **a** Line graph showing the effect of P4 (0 or 1 mM) with ( +) or without (-) rHuOVGP1 (50 mg/mL) on the level of tyrosine phosphorylation (pY) of p105 following 0 to 4 h of capacitation. **b** Histograms showing the relative intensity of tyrosine phosphorylation (pY) levels of p105 in sperm treated with P4 (0 or 1 mM) in the presence ( +) or absence (-) of rHuOVGP1 (50 mg/mL) after 2, 3, and 4 h of capacitation. Data are represented as relative intensity of pY + / − SEM; *n* = 7 patients; *p < 0.05; ns: statistically not significant

Our laboratory has previously reported that the tyrosine phosphorylation level of p105, a major human sperm protein, can be further enhanced in the presence of rHuOVGP1 at an optimal concentration of 50 mg/mL in the capacitating medium [27]. Based on these previous results and the current findings that P4 can increase the level of tyrosine phosphorylation of p105, we hypothesized that OVGP1 and P4 may work in synergy to enhance tyrosine phosphorylation of sperm p105. Western blot analysis was carried out to examine whether the increase in tyrosine phosphorylation observed in sperm treated with rHuOVGP1 and P4 each alone can be further enhanced when sperm are treated with rHuOVGP1 and P4 in combination. As shown in Fig. 5, the intensity of protein tyrosine phosphorylation was faintly detected at 0 h in non-capacitated sperm. The tyrosine phosphorylation level of p105 exhibited an increase in response to the presence of rHuOVGP1, similar to results previously reported [27], and in response to P4 compared to the control. Importantly, the strongest intensity of p105 was detected when P4 was used in combination with rHuOVGP1. Statistical analysis confirmed that the level of tyrosine phosphorylation of p105 was significantly increased with rHuOVGP1 alone after 2 and 3 h as well as with P4 alone after 2 and 4 h (Fig. 5c). Indeed, the level of tyrosine phosphorylation of p105 was substantially increased, respectively, at 2, 3, and 4 h after treatment with rHuOVGP1 and P4 in combination (Fig. 5c). The present results suggest that P4 and rHuOVGP1 may work in concert to increase tyrosine phosphorylation of p105 which could, in part, lead to enhancement of sperm capacitation.

## Discussion

Using recombinant human oviductin (rHuOVGP1) in conjunction with live cell imaging technique, we have been able to demonstrate, for the first time, an additional role of rHuOVGP1 which works in conjunction with P4 in enhancing sperm capacitation. Previous findings in our laboratory have demonstrated that rHuOVGP1 can bind to sperm to enhance tyrosine phosphorylation of sperm proteins, a biochemical hallmark of capacitation [27, 28]. However, the exact mode of action as how rHuOVGP1 enhances tyrosine phosphorylation of sperm proteins is not clearly understood and needs to be further investigated. Compiled evidence has shown that P4, another key component of the oviductal fluid that is secreted by cumulus cells surrounding the oocyte, induces Ca^2+^ influx through CatSper channels which are also located in the principal piece of the sperm tail and is an important aspect of capacitation [40–42]. As previous studies have implied a link between calcium signaling and tyrosine phosphorylation [36, 37], in the present study, we started out by examining if rHuOVGP1-enhanced tyrosine phosphorylation of sperm proteins could be mediated by an increase in intracellular Ca^2+^ via CatSper channels similar to P4 during sperm capacitation. We initially detected [Ca^2+^]_i_ in human sperm using the intracellular Ca^2+^ indicator Fluo3. Our results obtained with flow cytometry demonstrated that the level of intracellular Ca^2+^ gradually increased in human sperm at the beginning of capacitation, yet the concentration further increased when sperm were treated with rHuOVGP1 (Fig. 1a). Particularly, the presence of rHuOVGP1 in the capacitating medium was found to increase the level of [Ca^2+^]_i_ in a time- and dose-dependent manner at the beginning of human sperm capacitation (Fig. 1; Online Resource 1). This is the first report to show that rHuOVGP1 regulates [Ca^2+^]_i_ at the onset of sperm capacitation. We went further to investigate the influence of rHuOVGP1 on [Ca^2+^]_i_ focusing on the sperm tail. Live cell imaging was carried out where sperm were capacitated in the absence or presence of rHuOVGP1 followed by treatment with P4. We found that treating sperm with P4 alone increased [Ca^2+^]_i_ (Fig. 2a, b), which is supported by finding of previous studies showing that P4 induces Ca^2+^ influx through activating CatSper channels in sperm [40–42]. Moreover, there was an increasing trend in the level of [Ca^2+^]_i_ when sperm were treated with rHuOVGP1 (Fig. 2a–c: time-points a–c). Interestingly, there was a further increase in [Ca^2+^]_i_ following the addition of P4 in the capacitating medium where sperm cells were pre-treated with rHuOVGP1 and the resulting positive effects persisted over time (Fig. 2a–c: time-points d–g). Prior incubation of sperm with rHuOVGP1 appears to potentiate the positive effects of P4 on [Ca^2+^]_i_ in the sperm tail, presumably this glycoprotein works synergistically with P4 to upregulate [Ca^2+^]_i_. Furthermore, the present results indicate that the incubation period of sperm with rHuOVGP1 for potentiating the effect of P4 needs to be approximately 10 min or longer since the amplifying effect of P4-induced increase of [Ca^2+^]_i_ was not observed when sperm were incubated with rHuOVGP1 for 5 min (results not shown). These findings imply that, during sperm transport in the female reproductive tract, the interaction of sperm with the female oviductal secretions, of which OVGP1 is a major component, is particularly important in preparing the sperm to meet the oocyte for subsequent fertilization. Without sufficient interaction with OVGP1, although P4 released from cumulus cells can induce a surge of calcium influx that leads to sperm capacitation essential for acrosome reaction and subsequent fertilization, the level of [Ca^2+^]_i_ cannot be sustained and will diminish. On the contrary, sperm that come into contact with both OVGP1 and P4 maintain the increased level of [Ca^2+^]_I_ and likely maintain a greater potential to fertilize the oocyte.

Accumulating findings reported in literature have linked P4-induced Ca^2+^ influx in human sperm to CatSper channels [40–42]. Results obtained in the present study supported and extended the previous findings by demonstrating that treatment of human sperm with rHuOVGP1 can further enhance and sustain the positive effects of P4 on [Ca^2+^]_i_ in vitro. To examine if CatSper channels play a role in the increased in influx of [Ca^2+^]_i_ caused by rHuOVGP1, we used a specific inhibitor for CatSper channels, HC-056456, and performed live cell imaging to examine if rHuOVGP1 increases [Ca^2+^]_i_ by way of the CatSper channels. HC-056456 was used at both concentrations of 3 and 10 mM because previous research demonstrated that the use of HC-056456 at these concentrations was found to be an effective blocker of CatSper channels [43, 44]. As anticipated and based on reports in the literature, P4 was unable to induce an influx of Ca^2+^ when CatSper channels were blocked by the inhibitor (Fig. 3). Inhibition of CatSper channels by HC-056456 also hindered the effects of rHuOVGP1 in enhancing [Ca^2+^]_i_ that we observed earlier. Taken together, the present results indicate that OVGP1 increases [Ca^2+^]_i_ during capacitation at least, in part, through CatSper channels located in the sperm tail.

A time-dependent increase in protein tyrosine phosphorylation of sperm proteins is associated with capacitation [45]. Hence, we investigated whether P4 could enhance sperm capacitation by increasing tyrosine phosphorylation of sperm protein p105, similar to our previous findings showing that rHuOVGP1 can enhance tyrosine phosphorylation of p105 [27, 28]. We conducted a dose response experiment of P4 (0 nM, 1 nM, 10 nM, 100 nM, 1 mM, or 10 mM) on tyrosine phosphorylation of sperm p105 over a time course of 4 h. Western blot analysis revealed that the level of tyrosine phosphorylation of p105 was increased in sperm incubated in capacitating medium in the presence of higher concentrations (100 nM, 1 mM, and 10 mM) of P4 (Fig. 4a, b). These results are consistent with previous findings by Sagare-Patil and co-workers [37] where 1–10 mM of P4 were found to increase the level of tyrosine phosphorylation of sperm proteins over the first hour of capacitation. Our results also showed that the level of tyrosine phosphorylation of p105 was significantly increased in sperm capacitated in the medium containing 100 nM of P4 after 3 h (Fig. 4c). However, a significantly greater increase in the tyrosine phosphorylation level of p105 was observed in sperm capacitated in the medium containing 1 mM of P4 after 4 h (Fig. 4c). Altogether, the present results demonstrated that P4 alone can increase the tyrosine phosphorylation level of p105 in a dose- and time-dependent manner (Fig. 4; Online Resource 3). As the tyrosine phosphorylation level of p105 has been found previously to be under the influence of rHuOVGP1 [27] and now shown in the present study to be under the influence of P4, we were curious of their influence on tyrosine phosphorylation when used in combination. Western blot analysis showed an increase in tyrosine phosphorylation of p105 when sperm were capacitated in the presence of rHuOVGP1 alone or P4 alone as compared to the untreated cells (Fig. 5). However, the level of tyrosine phosphorylation was found to be further enhanced when sperm were capacitated in the medium containing both rHuOVGP1 and P4, with a peak intensity of tyrosine phosphorylation at 3 h of capacitation (Fig. 5). Thus, the present results clearly demonstrated that tyrosine phosphorylation of sperm protein p105 can be further enhanced when the capacitation medium is supplemented with both rHuOVGP1 and P4. A recent study carried out in our laboratory revealed that the sperm protein, p105, could be A-kinase anchoring protein 3 (AKAP3) [28], a prominent tyrosine phosphorylated protein associated with the fibrous sheath of the sperm tail [46, 47]. AKAP3 binds to the regulatory subunits of protein kinase A (PKA) and confines PKA to sub-cellular compartments of the sperm cell to regulate the cAMP-PKA pathway, a key signaling pathway for sperm capacitation [48–50]. Tyrosine phosphorylation of AKAP3 is associated with an increased binding of the protein to PKA, resulting in the recruitment and activation of PKA to the fibrous sheath, ultimately, leading to enhanced sperm motility [49].

Collectively, our experimental results demonstrated that rHuOVGP1 and P4 function most effectively in combination to enhance human sperm capacitation through increasing [Ca^2+^]_i_ and protein tyrosine-phosphorylation. It is now known that P4 acts on the CatSper channel via a membrane endocannabinoid signaling pathway through the α/β hydrolase domain–containing protein 2 (ABHD2) that serves as a sperm P4 receptor [41]. At rest, CatSper channels are inhibited by the endocannabinoid 2-arachidonoylglycerol (2-AG) in the membrane of the sperm tail. Binding of P4 to ABHD2 degrades 2-AG thus abolishing the inhibition and activating CatSper channels leading to Ca^2+^ influx that is essential for the hyperactivation of the sperm flagellum [41]. The activation of CatSper channels by P4 and other biological compounds has been eloquently reviewed recently by Rahban and Nef [51]. The interplay between P4 and CatSper as well as other factors during capacitation that leads to protein tyrosine phosphorylation of sperm proteins has also been thoroughly reviewed [52] and, therefore, will not be further discussed here. Although the mechanism by which rHuOVGP1 increases the influx of Ca^2+^ by way of CatSper channels remains elusive, based on the results obtained in the present study, we propose that OVGP1 may work in concert with P4 to upregulate [Ca^2+^]_i_ by way of CatSper channels in the sperm tail at the onset of capacitation. We are hypothesizing that OVGP1 binds to a plasma membrane receptor of the principal piece of the human sperm tail. Binding of OVGP1 to its receptor might start the initial abolition of inhibition of the CatSper channel that could prepare for the subsequent action of P4. The resulting increase of influx of [Ca^2+^]_i_, in turn, leads to the downstream event of tyrosine phosphorylation of sperm proteins and enhancement of sperm capacitation and fertilizing competence of sperm. The true nature of a putative OVGP1 receptor and its mechanism of action remain elusive and need further investigations.

Although the present study focused on investigating the effects of OVGP1 and P4 on [Ca^2+^]i in the sperm tail, it is worth mentioning that a significant increase in [Ca^2+^]i was also detected in the sperm head in the presence of both rHuOVGP1 and P4. It is now known that the calcium influx is an absolute requirement for acrosome reaction to occur in all invertebrates and mammalian sperm [53]. Whereas CatSper is restricted predominantly or completely in the principal piece of the sperm tail in mice [31] and in humans [54], the major Ca^2+^ influx channels in the sperm head plasma membrane responsible for calcium entry and intracellular calcium ion levels are voltage-activated and voltage-dependent calcium channels [53]. Results of the present study showed that, similar to what was observed in the sperm tail, OVGP1 appears to potentiate the effect of P4 in increasing [Ca^2+^]i in the sperm head. The mechanism of action of OVGP1 in regulating Ca^2+^ in the sperm head and its possible involvement in acrosome reaction need further exploration.

According to the WHO laboratory manual for the examination and processing of human semen, the lower motility limit for normozoospermic samples is 40% [38]. Due to the limited availability of patient samples in the present study, a small portion of experiments that we conducted involved the use of samples with motility lower than normal. We separated our live cell imaging results presented earlier in Fig. 3 into low motility (≤ 40% motility) and normal motility (> 40% motility) groups. It appears that the influence of rHuOVGP1 on amplifying the positive effects of P4 on [Ca^2+^]_i_ in human sperm tail is more pronounced in sperm with low motility compared to sperm with normal motility (see chart in Online Resource 4). It will also be of interest, in the future, to examine the effects of rHuOVGP1 on [Ca^2+^]_i_ specifically in sperm with low motility using a larger sample size as this could further validate the benefits of rHuOVGP1 for patients with mild male factor infertility. Since poor sperm motility can be a cause of male factor infertility, addition of rHuOVGP1 in the capacitating medium, along with P4 secreted by cumulus cells surrounding the oocyte, could enhance the sperm with low motility to move efficiently. This could lead to improved sperm-egg binding and higher fertilization rate in the conventional in vitro fertilization (IVF) procedure.

## Conclusion

The present investigation aimed at gaining a better understanding of the function of human OVGP1 in enhancing sperm capacitation. The present research revealed that rHuOVGP1 increases [Ca^2+^]_i_ in human sperm at the beginning of capacitation. Furthermore, treatment of sperm with rHuOVGP1 further increases and sustains the level of [Ca^2+^]_i_ in the sperm tail following the addition of P4. Inhibition of CatSper channels impedes the effects of rHuOVGP1 on [Ca^2+^]_i_ in human sperm. This suggests that OVGP1 may, in part, influence Ca^2+^ influx through the CatSper channels. Lastly, we have demonstrated that P4 alone can increase tyrosine phosphorylation of a major human sperm protein, yet yield further increase when rHuOVGP1 and P4 are used in combination. The current findings could be of significance to the field of infertility as a better understanding of the function of OVGP1 could improve the success rates of conventional IVF procedures by supplementing the capacitating medium currently used in fertility clinics with rHuOVGP1. Improving fertility treatments for infertile couples remains a high priority in reproductive medicine. It is hoped that the positive effects on sperm capacitation resulting from OVGP1 accompanied by the secretion of P4 by cumulus cells encircling the post-ovulatory oocyte can open new avenues for further investigation into the mechanism that regulate human OVGP1in sperm function.

## Supplementary information

Below is the link to the electronic supplementary material.

Supplementary file1 rHuOVGP1 increases [Ca^2+^]_i_ in human sperm at the beginning of capacitation. Table shows the level of Fluo3 median intensity from the flow cytometry analysis of [Ca^2+^]_i_ in sperm. Following 3 min (shown as -200 to -20 s) of baseline fluorescence reading, rHuOVGP1 (0, 25, 50, 75, and 100 mg/mL) was added, respectively, to the cell suspension (time = 0 s), and the data were acquired for a further 10 min. The level of Fluo3 median intensity of each concentration of rHuOVGP1 (0, 25, 50, 75, and 100 mg/mL) over time was compared to that prior to the addition of rHuOVGP1 at -45 s. n = 7 patients; * p < 0.05, ** p < 0.01, *** p < 0.001, **** p < 0.0001 (DOCX 492 KB)

Supplementary file2 The effects of rHuOVGP1 on the level of [Ca^2+^]_i_ in human sperm heads following treatment with P4. a: Representative images of calcium live cell imaging experiments with or without rHuOVGP1 (100 mg/mL) treatment. High magnifications of sperm cells indicated by blue (pre-P4) and red (post-P4) dashed boxes are shown on the right side of the figure with regions of interest (ROIs) of sperm heads outlined in yellow. Sperm cells were imaged for 1 min in BWW to obtain baseline measurements, followed by subsequent imaging the same cells treated with or without rHuOVGP1 (100 mg/mL) for 10 min and progesterone (P4; 1 mM) for 5 min. b: Line graph of the relative mean fluorescent intensity of Fluo3 from the live cell imaging analysis of [Ca^2+^]_i_ in sperm heads treated with or without rHuOVGP1 (100 mg/mL) followed by the treatment with P4 (1 mM). Data are represented as fold of change in fluorescent labeling ± SEM; n = 6 patients; * p < 0.05 (DOCX 816 KB)

Supplementary file3 P4 enhances the level of tyrosine phosphorylation of human sperm protein. Table shows the effect of different P4 concentrations (0, 1 nM, 10 nM, 100 nM, 1 mM, or 10 mM) on the level of tyrosine phosphorylation of p105 from Western blot analysis following 0 to 4 h of capacitation. The relative intensity of tyrosine phosphorylation of p105 for treatment with each concentration of P4 over time (1-, 2-, 3-, and 4-h) compared to that of 0-h capacitation. n = 6 patients; * p < 0.05, ** p < 0.01, *** p < 0.001 (DOCX 220 KB)

Supplementary file4 Segregation of results for normal and low motility sperm treated with rHuOVGP1 followed by subsequent treatment with P4. Line graph of the relative mean fluorescent intensity of Fluo3 from the live cell imaging analysis of [Ca^2+^]_i_ in sperm tail treated with or without rHuOVGP1 (100 mg/mL) followed by the treatment with P4 (1 mM). Data are represented as fold of change in fluorescent labeling ± SEM; control group n = 6 patients; sperm with > 40% motility n = 4 patients; sperm with ≤ 40% motility; n = 2 patients (DOCX 270 KB)
