# Supplementary figures and images for: Recombinant human OVGP1 increases intracellular calcium and further potentiates the effects of progesterone on human sperm
Source: J Assist Reprod Genet. 2022 Aug 16;39(10):2287–301. doi: 10.1007/s10815-022-02591-0 (PMC9596666; doi:10.1007/s10815-022-02591-0)

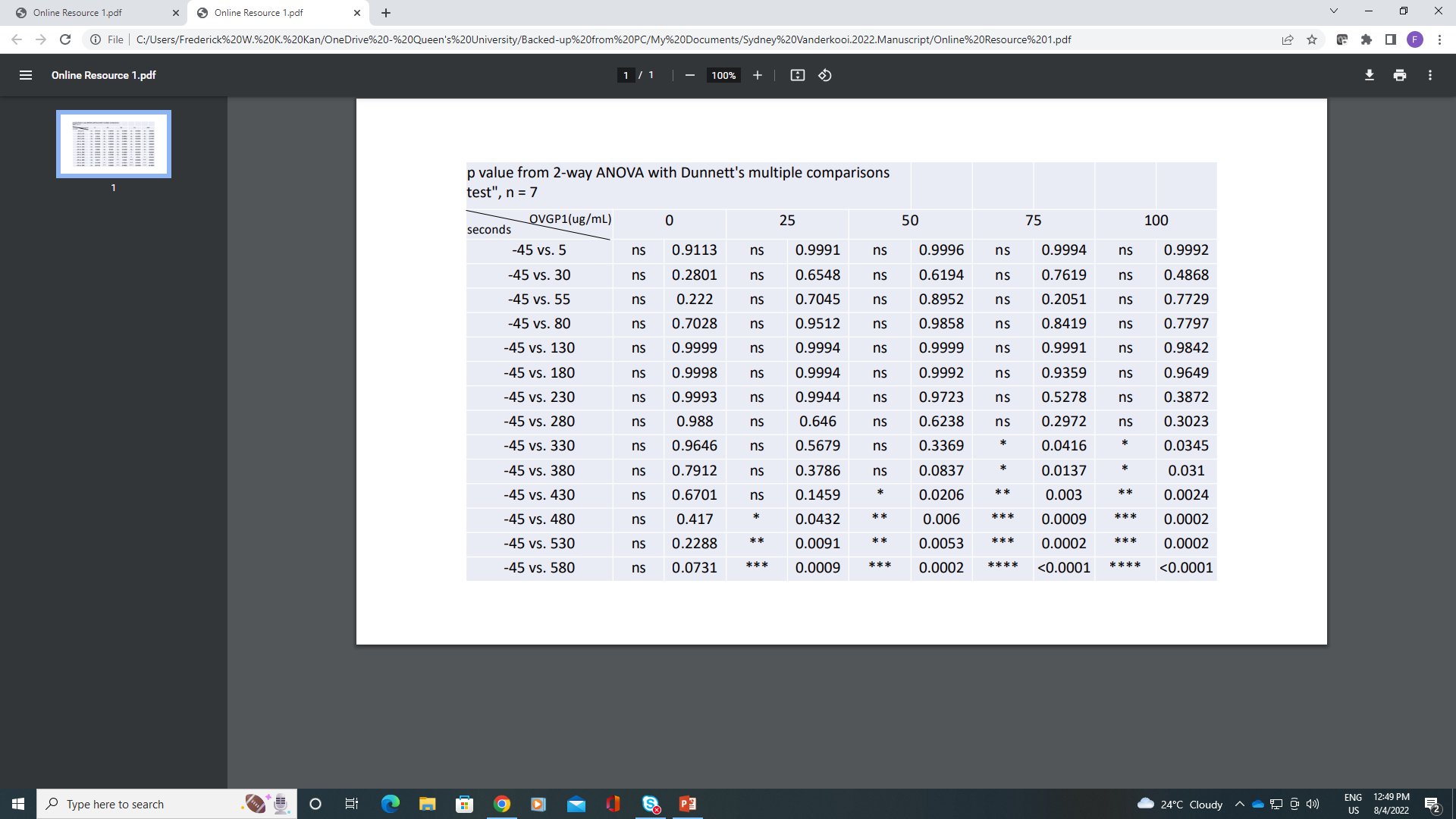
Online Resource 1

Supplement: Supplementary file 1 — Supplementary file1 rHuOVGP1 increases [Ca2+]i in human sperm at the beginning of capacitation. Table shows the level of Fluo3 median intensity from the flow cytometry analysis of [Ca2+]i in sperm. Following 3 min (shown as -200 to -20 s) of baseline fluorescence reading, rHuOVGP1 (0, 25, 50, 75, and 100 mg/mL) was added, respectively, to the cell suspension (time = 0 s), and the data were acquired for a further 10 min. The level of Fluo3 median intensity of each concentration of rHuOVGP1 (0, 25, 50, 75, and 100 mg/mL) over time was compared to that prior to the addition of rHuOVGP1 at -45 s. n = 7 patients; * p < 0.05, ** p < 0.01, *** p < 0.001, **** p < 0.0001 (DOCX 492 KB) [file 10815_2022_2591_MOESM1_ESM.docx]

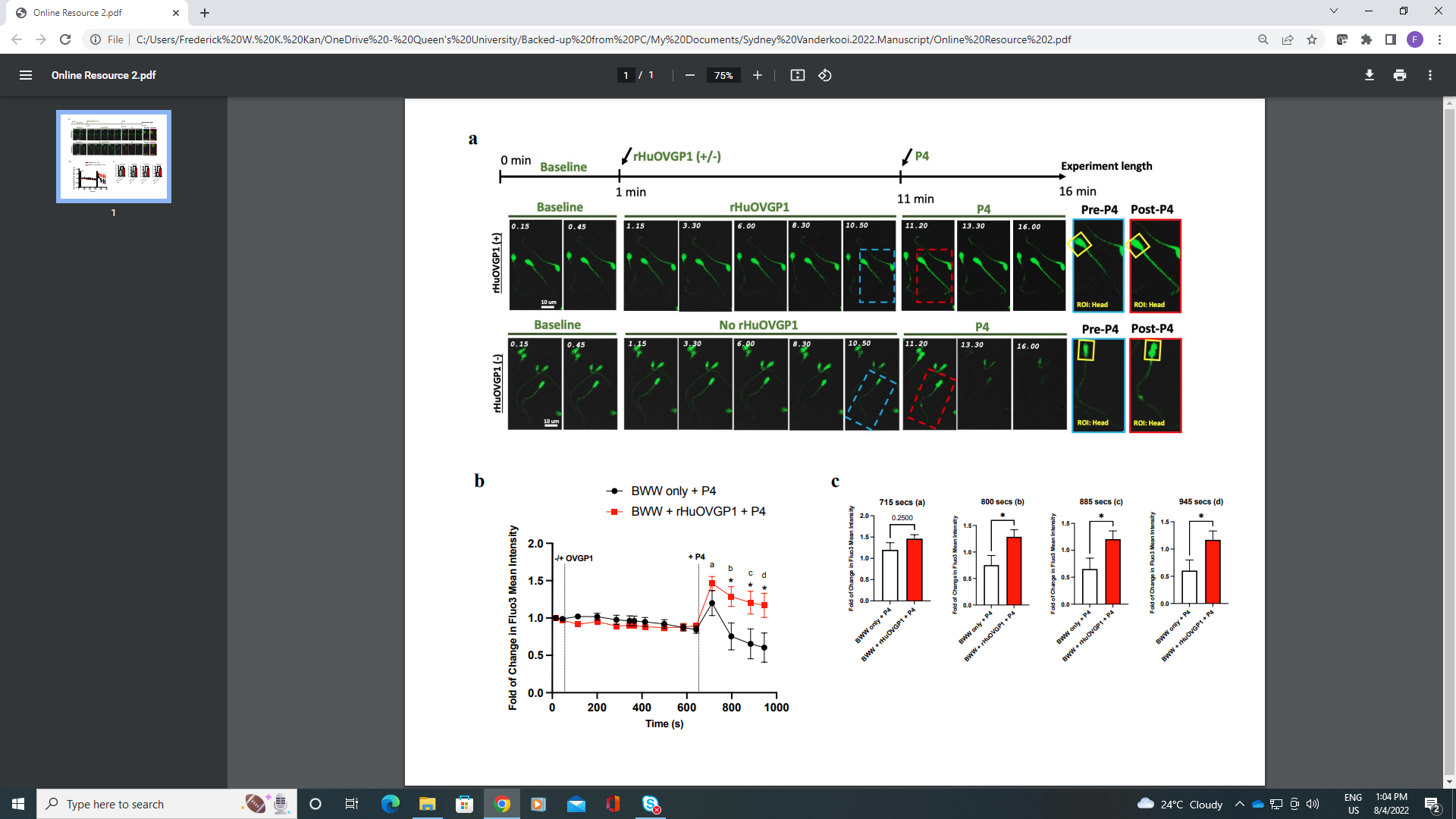
Online Resource 2

Supplement: Supplementary file 2 — Supplementary file2 The effects of rHuOVGP1 on the level of [Ca2+]i in human sperm heads following treatment with P4. a: Representative images of calcium live cell imaging experiments with or without rHuOVGP1 (100 mg/mL) treatment. High magnifications of sperm cells indicated by blue (pre-P4) and red (post-P4) dashed boxes are shown on the right side of the figure with regions of interest (ROIs) of sperm heads outlined in yellow. Sperm cells were imaged for 1 min in BWW to obtain baseline measurements, followed by subsequent imaging the same cells treated with or without rHuOVGP1 (100 mg/mL) for 10 min and progesterone (P4; 1 mM) for 5 min. b: Line graph of the relative mean fluorescent intensity of Fluo3 from the live cell imaging analysis of [Ca2+]i in sperm heads treated with or without rHuOVGP1 (100 mg/mL) followed by the treatment with P4 (1 mM). Data are represented as fold of change in fluorescent labeling ± SEM; n = 6 patients; * p < 0.05 (DOCX 816 KB) [file 10815_2022_2591_MOESM2_ESM.docx]

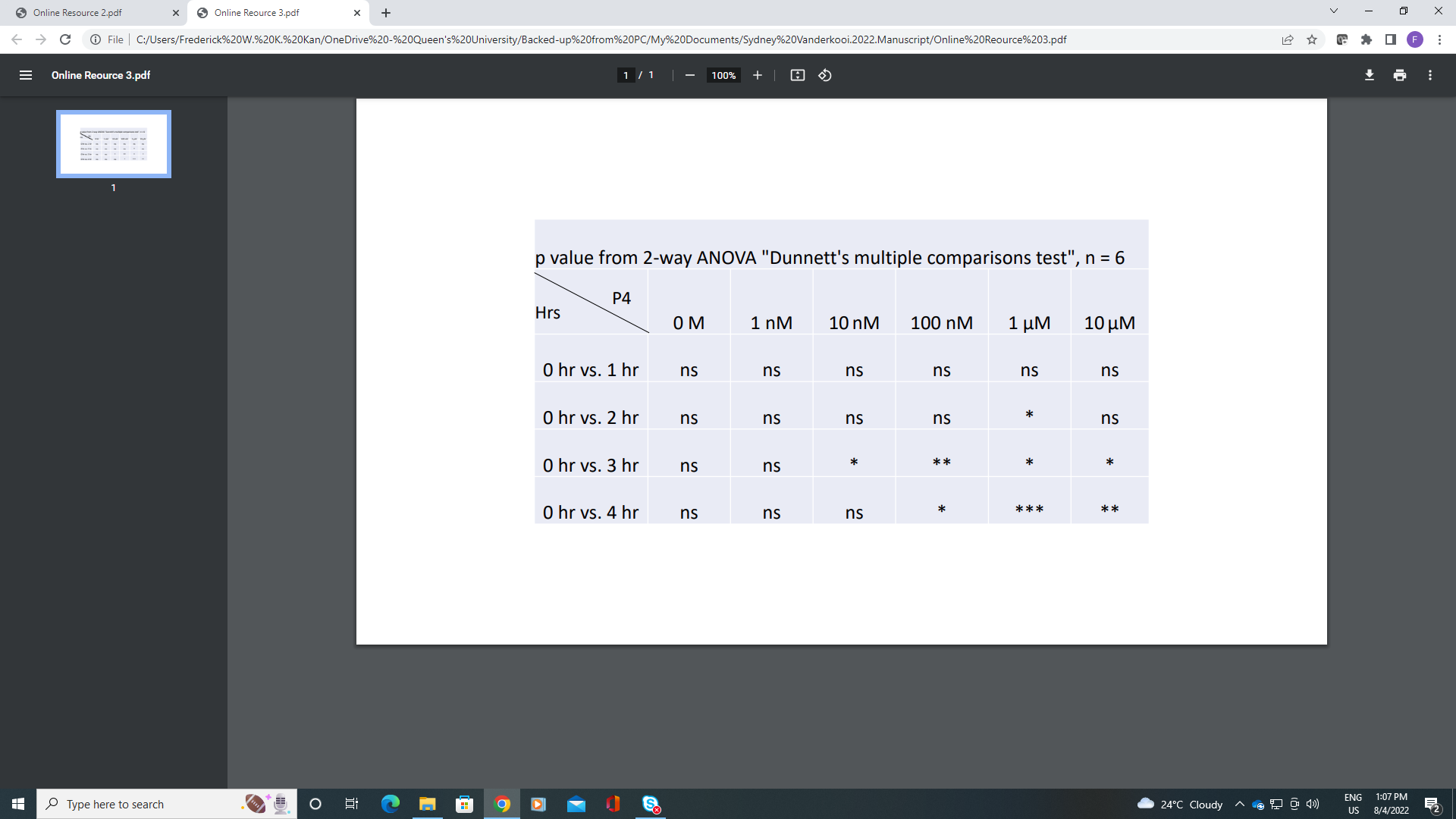
Online Resource 3

Supplement: Supplementary file 3 — Supplementary file3 P4 enhances the level of tyrosine phosphorylation of human sperm protein. Table shows the effect of different P4 concentrations (0, 1 nM, 10 nM, 100 nM, 1 mM, or 10 mM) on the level of tyrosine phosphorylation of p105 from Western blot analysis following 0 to 4 h of capacitation. The relative intensity of tyrosine phosphorylation of p105 for treatment with each concentration of P4 over time (1-, 2-, 3-, and 4-h) compared to that of 0-h capacitation. n = 6 patients; * p < 0.05, ** p < 0.01, *** p < 0.001 (DOCX 220 KB) [file 10815_2022_2591_MOESM3_ESM.docx]

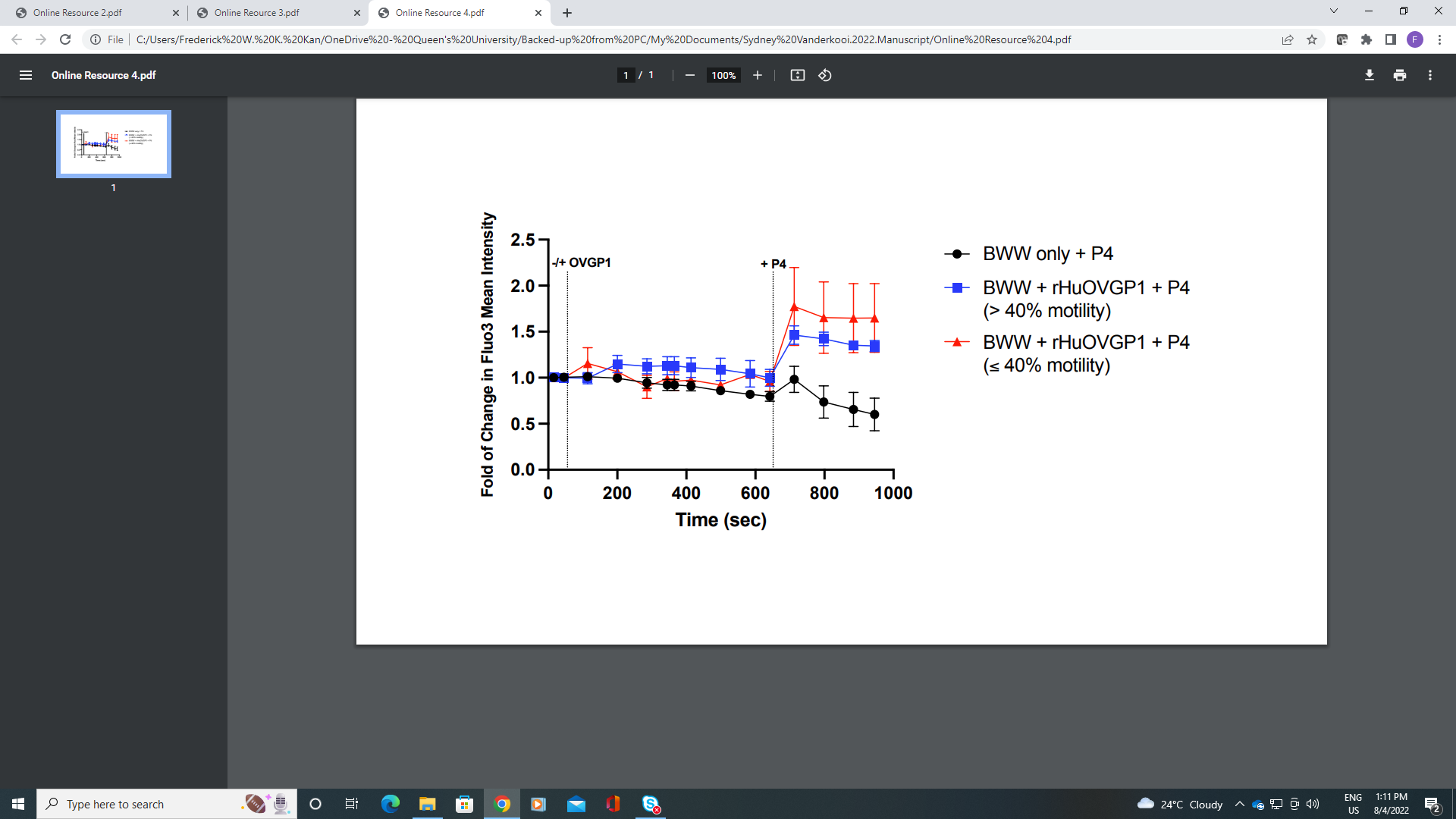
Online Resource 4

Supplement: Supplementary file 4 — Supplementary file4 Segregation of results for normal and low motility sperm treated with rHuOVGP1 followed by subsequent treatment with P4. Line graph of the relative mean fluorescent intensity of Fluo3 from the live cell imaging analysis of [Ca2+]i in sperm tail treated with or without rHuOVGP1 (100 mg/mL) followed by the treatment with P4 (1 mM). Data are represented as fold of change in fluorescent labeling ± SEM; control group n = 6 patients; sperm with > 40% motility n = 4 patients; sperm with ≤ 40% motility; n = 2 patients (DOCX 270 KB) [file 10815_2022_2591_MOESM4_ESM.docx]
